# Spectrums of opportunistic infections in HIV-Infected patients in referral hospital of Setif (Algeria)

**DOI:** 10.1186/1471-2334-14-S2-P50

**Published:** 2014-05-23

**Authors:** A Ouyahia, M Rais, A Gasmi, W Guenifi, S Mechakra, A Lacheheb

**Affiliations:** 1 Ferhat Abbes University, faculty of medicine, Setif, Algeria

## Background

The clinical course of HIV/AIDS and pattern of opportunistic infections vary from patient to patient and from country to country. in this report we describe the clinical and laboratory profiles of different opportunistic infections (OIs) among 266 immunocompromised patients admitted to a referral hospital in the eastern of Algeria.

## Methods

Between January 2003 and November 2013, 266 HIV infected patients were admitted to our center. Clinical data about patients were collected retrospectively from standardized HIV/AIDS forms filled at admission.

## Results

Out of 266 patients, 65% were male, the mean age was 37 years and the most frequent route of transmission was heterosexual (86%). 60 % were admitted with obvious clinical signs and symptoms, whereas 40% were asymptomatic, and they were diagnosed through screens or check-up tests. The most frequent clinical symptoms on first admission were oral candidiasis (66.4%), herpes zoster virus infection (18.6%), tuberculosis (16.7%), Pneumocystis jirovecii pneumoniae (12 %), weight loss (10 %), persistent generalized lymphadenopathy (3.5 %), , malignancies (3.2 %), chronic fever (2.5%). We found the low prevalence of AIDS-defining illnesses in central neural system in this study, including progressive multifocal leukoencephalopathy (1.8%), cerebral toxoplasmosis (3 %), cryptococcal meningitis (2.3%).

Median viral load was 120000cp/mL. Screening tests were conducted most frequently during blood donations (20%), before surgeries (13 %) and check-ups (6 %).

## Conclusion

Algeria is among low prevalence countries in the world for HIV/AIDS. Clinical profile of our patients is similar to the developed countries. More than half of the patients diagnosed at late stages of the disease. It highlights the need for early screening and also the need to increase awareness in healthcare providers, in order to improve decisions regarding prophylaxis for prevention and appropriate therapeutic intervention.

